# Whole-Body Vibration Training Increases Myocardial Salvage Against Acute Ischemia in Adult Male Rats

**DOI:** 10.5935/abc.20180252

**Published:** 2019-01

**Authors:** Shahnaz Shekarforoush, Mohammad Reza Naghii

**Affiliations:** 1 Islamic Azad University, Arsanjan Branch, Fars - Iran; 2 Sport Physiology Research Center, Baqiyatallah University of Medical Sciences, Teerã - Iran

**Keywords:** Rats Wistar, Body Composition, Vibration, Osteoporosis/prevention and control, Blood Viscosity, Ischemia, Cardiovascular Diseases, Ischemic Preconditioning

## Abstract

**Background:**

Whole body vibration training (WBV) is a new training program, which is safe and effective. It can be followed by the public. However, data on the safety and efficacy of vibration on myocardial ischemia reperfusion (IR) injury are lacking.

**Objective:**

To examine the effect of WBV on the tolerance of the myocardium to acute IR injury in an experimental rat model.

**Methods:**

Twenty-four male Wistar rats were divided into control and vibration groups. Vibration training consisted of vertical sinusoidal whole body vibration for 30 min per day, 6 days per week, for 1 or 3 weeks (WBV1 and WBV3 groups, respectively). All the rats were submitted to myocardial IR injury. Myocardial infarct size and ischemia-induced arrhythmias were assessed. Differences between variables were considered significant when p < 0.05.

**Results:**

No differences were observed between the groups regarding the baseline hemodynamic parameters. Infarct size was smaller in the experimental group (control, 47 ± 2%; WBV1, 39 ± 2%; WBV3, 37 ± 2%; p < 0.05, vs. control). Vibration produced a significant decrease in the number and duration of ventricular tachycardia (VT) episodes compared to the control value. All ventricular fibrillation (VF) episodes in the vibration groups were self-limited, while 33% of the rats in the control group died due to irreversible VF (p = 0.02).

**Conclusion:**

The data showed that vibration training significantly increased cardiac tolerance to IR injury in rats, as evidenced by reduction in the infarct size and cardiac arrhythmias, and by facilitating spontaneous defibrillation.

## Introduction

Whole body vibration training (WBV) has been recently proposed as an exercise training method with a potential for improving body composition and preventing osteoporosis and bone mass loss.^1^ In recent years, some studies have shown that WBV may be a beneficial training mode in patients with multiple sclerosis,^2^ type 2 diabetes,^3^ chronic obstructive pulmonary disease,^4^ and heart transplant recipients.^5^ The effects of WVB on the cardiovascular system were investigated in a number of published studies. Decreased arterial stiffness after WBV can reduce the risk of cardiovascular disease.^6^^,^^7^ An experiment conducted by Robbins et al.^8^ showed a significant increase in blood flow velocity with no significant changes in heart rate, blood pressure or peripheral skin temperature. Increased muscle blood volume and blood flow velocity after vibration exercise were attributed mainly to the effect of vibrations in reducing blood viscosity and increasing its velocity through the arteries.^9^ These findings indicate that WBV may represent a mild form of exercise for the cardiovascular system.^10^

Cardiovascular disease (CVD), which is induced by ischemia, is the leading cause of death worldwide. Restoration of blood flow, after a period of ischemia, can elicit pathological processes that exacerbate injury due to the ischemia itself.^11^ Preconditioning describes a pretreatment or premaneuver that is able to adapt the myocardium to ischemic stress. We have demonstrated some preconditioning interventions in previous experiments, reducing infarct size and arrhythmias.^12^^,^^13^ The cardioprotective effect of exercise preconditioning was reported as a reduction in infarct size in previous studies.^14^^,^^15^ Accumulating evidence indicates that both short-term (i.e. 1-5 days) and long-term (i.e. weeks to months) exercise can protect the heart during an ischemia-reperfusion (IR) insult. While much is currently known about exercise preconditioning, to our knowledge, the effect of whole-body vibration on IR injury has not been investigated. Thus, the purpose of this study was to determine whether the vibration exercise would be able to reduce infarct size and arrhythmia during IR injury in an experimental rat model.

## Methods

Male Wistar rats weighing 250 to 300 g (10-12 weeks old) were obtained from the animal house of Shiraz University of Medical Sciences and housed under standard conditions, with free access to food and water. The investigation was approved by the University Ethics Committee in accordance with the Guide for the Care and Use of Laboratory Animals.

### Experimental designs

A total of 24 rats were randomly assigned to 1 of 3 treatment groups (control vs. two experimental groups) by picking numbers out of a hat. The sample size (n) was established based on studies that evaluated the effects of exercise against myocardial IR injury.^16^^,^^17^ Animals in the vibration groups were placed in a compartment attached to a vibration platform (Crazy Fit Massager/Model: YD 1002, Union Brilliant Group Co., LTD, Fujian, China). The vibration training consisted of a 5-min cycle on day 1, followed by an extra 5-min cycle each time for the next five sessions in the first week and then each rat was exposed to vertical sinusoidal vibration for 30 min per session (3 × 10 min cycles), 6 days a week for one week (WBV1 group) or 3 weeks (WBV3 group). The animals were given 1-2 min rest break between the cycles. The vibration was performed at mode 1 with amplitude of 1-10 mm and at a frequency of 10-50 Hz. The speed of mode 1 in each cycle increased gradually and then decreased with the same trend within each time period. The control animals remained in their cages and were placed over the vibration platform, without vibration treatment. Each training session was performed between 8.30-10.00 A.M.^18^

### Surgical procedure

The protocol used has been thoroughly described in detail in our previous publication.^12^ Briefly, 24 hours after the last training session, the animals were anesthetized and ventilated with room air enriched with oxygen at a rate of 70 breaths per min. A standard limb lead II electrocardiogram was monitored and recorded throughout the experiment. Catheters were inserted into the left carotid artery and tail vein for monitoring of blood pressure and infusion of Evans blue solution, respectively. After the thoracotomy, a 6-0 silk suture was passed around the left anterior descending coronary artery (LAD). Following a stabilization period of 20 min, the LAD was occluded for 30 min of ischemia and released for 120 min of reperfusion. Rectal temperature was continuously monitored and maintained at 37 ± 0.5ºC.

### Determination of infarct size and area at risk

At the end of reperfusion, the LAD was reoccluded and 1 mL of 2% solution of Evans Blue dye (Sigma, St. Louis, MO) was injected into the tail vein to identify the non-perfused area, also known as area at risk (AAR), from the perfused area. The rats were then killed with a pentobarbital overdose and their hearts were excised and frozen for one hour. The atria and right ventricle were removed, and the left ventricle was cut into transverse slices of 2 mm thickness from the apex to the base. Tissue samples were then incubated with a 1% solution of 2,3,5 triphenyltetrazolium chloride (Sigma)] for 20 min at 37ºC, and subsequently fixed in 10% phosphate-buffered formalin for one hour. Viable myocardium was stained red by triphenyltetrazolium chloride, whereas necrotic myocardium appeared as pale yellow. In each slice, areas at risk and infarcted areas were determined by computerized planimetry using an image analysis software (Image Tool, University of Texas, San Antonio, TX). Infarct size (IS) was expressed as percentage of the AAR (IS/AAR).^12^

### Assessment of ventricular arrhythmias

Ischemia-induced ventricular arrhythmias were determined in accordance with the Lambeth conventions^19^ including ventricular ectopic beat as premature ventricular complexes (PVC), ventricular tachycardia (VT) as a run of four or more consecutive ventricular premature beats at a rate faster than the resting sinus rate, and ventricular fibrillation (VF) as a signal for which individual QRS deflection can no longer be distinguished from one another. Complex forms (bigeminy and salvos) were added to PVC count and not analyzed separately. In order to determine the incidence of VT and VF, they were recorded as either occurring or not occurring during the first 30 min of ischemia in each group.

### Statistical analyses

Unless stated otherwise, the results were expressed as Mean ± SD. All data were processed with the SPSS 16.0 statistical package for Windows version. The normality of distributions was verified by the Kolmogorov-Smirnov test. Fisher exact test (Chi-square) was used to analyze the incidence of VT and VF. Analysis of baseline, ischemia, and reperfusion HR and BP was done by repeated measures analysis of variance (ANOVA). The other data were analyzed using one-way ANOVA and then significant differences were examined by Tukey’s post-hoc test. Differences between the groups were considered significant at a level of p < 0.05.

## Results

### Hemodynamic parameters

Table 1 summarizes the hemodynamic data. There were no significant differences at baseline values for heart rate (HR) and mean arterial blood pressure (MBP) among the groups. Ischemia caused a marked reduction in blood pressure without any significant effect on the HR in the groups. MBP was nearly restored to the baseline level during the reperfusion period.

**Table 1:** Hemodynamics parameters in the experimental groups

Group	Baseline		Ischemia		Reperfusion	
	HR	MBP	HR	MBP	HR	MBP
Cont	346 ± 48	113 ± 21	349 ± 51	100 ± 15* (0.04)	361 ± 44	107 ± 17
WBV1	373 ± 41	114 ± 7	385 ± 25	97 ± 8**(0.001)	383 ± 26	107 ± 13
WBV3	376 ± 26	107 ± 15	372 ± 18	91 ± 7* (0.01)	378 ± 27	108 ± 15
p-value	0.282	0.670	0.130	0.267	0.399	0.996

Note: Data presented as mean ± SD (P-value). HR: heart rate; MBP: mean arterial blood pressure. Cont: control, WBV1: whole body vibration training for one week, WBV3 = whole body vibration training for 3 weeks.

* p < 0.05,

** p < 0.01 compared to baseline value.

### Infarct size

**Figure 1:**
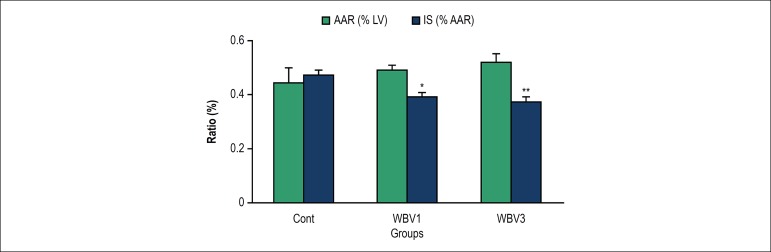
Infarct size (IS) and area at risk (AAR) following 30-min of ischemia and 120-min of reperfusion in rats. LV: left ventricle; * p < 0.05 and ** p < 0.01 compared with the control group. Cont: control; WBV1: whole body vibration training for one week; WBV3: whole body vibration training for 3 weeks.

Figure 1 shows AAR and IS following 30 min of regional ischemia and 120 min of reperfusion. There was no marked difference in AAR/LV ratio among the groups (p = 0.92). Infarct size was 47 ± 5% in the control group. WBV1 or WBV3 resulted in a smaller infarct size, i.e. 39 ± 5% and 37 ± 5% (p = 0.047 and p = 0.009 vs. the controls), respectively.

### Ischemia-induced arrhythmias

Table 2 represents the number of PVC, VT and VF episodes and their duration during the 30-min ischemic period. The arrhythmias occurred after approximately 5-7 min of ischemia. The number of PVC decreased non-significantly in the experimental groups (p = 0.702). Vibration produced a significant decrease in the number and duration of VT episodes compared to the control value. The mean duration of reversible VF in the WBV3 group was reduced from 32.3 ± 19.4 s in the control group to 13.7 ± 10.3 s (as a non-significant trend). Although the longest VF episodes in the vibration groups lasted as much as 116 s, all VF episodes were self-limited. However, the longest observed non-fatal VF episode in the control group was 87 s. and 33% of the rats died due to irreversible VF (p = 0.02). The occurrence (% incidence per group) of VT during the 30-min ischemia was 100, 100 and 88% (p = 0.35) and the occurrence of VF was 75, 63 and 50% in the control, WBV1 and WBV3 groups, respectively (p = 0.58).

**Table 2:** Incidence and duration of ventricular arrhythmias during 30 min of ischemia

Groups	PVC (n)	VT		VF	
		Episodes	Duration	Episodes	Duration
Cont	283 ± 50	31 ± 4	70 ± 14	2.0 ± 0.9	32.3 ± 19.4
WBV1	271 ± 32	17 ± 1*	54 ± 19	2.3 ± 0.8	33.2 ± 17.9
WBV3	229 ± 55	13 ± 3**	12 ± 4*	1.0 ± 0.6	13.7 ± 10.3
p-value	0.702	0.002	0.018	0.559	0.475

PVC: premature ventricular complexes; VT: ventricular tachycardia; VF: ventricular fibrillation; WBV1: whole body vibration training for one week; WBV3: whole body vibration training for 3 weeks

The numbers of premature ventricular complexes (PVC), the ventricular tachycardia (VT) and ventricular fibrillation (VF) episodes and duration are shown as means ± SEM. * p < 0.05 and ** p < 0.01 compared with the control group. Cont: control; WBV1: whole body vibration training for one week; WBV3: whole body vibration training for 3 weeks.

## Discussion

There are three main findings of the present study. First, WBV caused a significant decrease in IS following 30 min of ischemia and 120 min of reperfusion. Second, WBV had a protective effect on ischemia-induced arrhythmia. Third, all VF episodes were self-limited in the vibration groups, so the vibration improved arrhythmia-related mortality.

There are conflicting results regarding the effect of WBV on BP and HR. Performing dynamic exercise on a vertical vibration platform (30-35 Hz, 2 mm) for 12 weeks resulted in decreased systolic blood pressure in patients suffering from type 2 diabetes.^20^ Figueroa et al.’s study showed that 6 weeks of WBV decreased systemic arterial stiffness and systolic blood pressure in young overweight/obese normotensive women.^7^ Unlike these results, it was demonstrated that one session of exercise with vibration increased systolic and diastolic blood pressure and stroke volume compared with exercise with no vibration in sedentary adults.^21^ In contrast, some researchers have reported that WBV had no effect on the systolic and diastolic blood pressure, which is similar to our study results.^6^^,^^8^^,^^22^ These conflicting results may be explained by the different experimental conditions, including duration of the treatment and possibly the heterogeneity of the health status of participants in the different studies.

The ischemia reperfusion model in the experimental animals provides an option to evaluate the occurrence of ischemia-induced arrhythmias and infarct size after an intervention. Posa et al. demonstrated that 6 weeks of voluntary exercise was protective against IR injury by reducing the myocardial infarct size.^23^ An important finding is that one-to-several days of exercise can also reduce myocardial damage due to IR injury.^24^ Studies have demonstrated that regular exercise increases antioxidant capacity in the heart, which can minimize oxidative stress following IR.^25^ During all sporting activities, externally-applied forces induce vibrations within the body tissues.^10^ WBV has been proposed as an efficient alternative to moderate intensity exercise.^26^

Although recent studies have suggested that WBV leads to improvements in numerous health outcomes, including bone mineral density,^27^ muscle strength, or cardiovascular fitness,^28^ no research has been performed so far to evaluate the effects on IR injury.

The present study demonstrated that WBV is able to reduce myocardial infarct size and ischemia-induced arrhythmia during IR injury in rats. In the course of myocardial infarction, ventricular arrhythmias such as VT and VF are the most important cause of mortality.^29^ There was no difference in the ratio of AAR/LV between the control and vibration animals, indicating that all animals suffered a comparable degree of ischemic area. Therefore, the reduction of infarct size and arrhythmia in vibration-treated animals was due to the effect of the training. There are two types of VF: a sustained VF (SVF) that never terminates spontaneously and requires electrical defibrillation and a transient VF (TVF) that terminates by itself and spontaneously reverts into a sinus rhythm. Although it was believed for many years that TVF appears only in small mammals (rats, guinea pigs and rabbits), no differences were found in cardiac muscle mass, heart rate and action potential duration between animals with TVF and those with SVF. Intercellular uncoupling during ischemia most likely due to an increase in the intracellular Ca^2+^ and H^+^ ions or a decrease in the intracellular cAMP may lead to SVF. Therefore, any defibrillating intervention should prevent intercellular uncoupling, most probably by increasing the intracellular concentration of cAMP, decreasing elevated [Ca^2+^]_i_ or preventing Ca^2+^ overload.^30^ The results of the present study suggested that all VF episodes were self-limited in the vibration groups. Thus, vibration training could reduce the risk of sudden death during ischemia, through both attenuation of the ischemia-induced arrhythmia and facilitation of spontaneous defibrillation. The exact mechanism of action by which vibration reduces the incidence of fatal VF episodes cannot be directly assessed by our study. However, the increased ventricular fibrillation threshold in trained hearts during acute regional ischemia was shown in previous studies.^31^ Additionally, exercise training has been reported to increase the levels of cAMP^32^ and to improve cardiomyocyte function and diastolic Ca^2+^control in rats with post-infarction heart failure.^33^^,^^34^ Several studies have also shown a positive correlation between infarct size and the occurrence of severe ventricular arrhythmias.^35^^,^^36^

Currently, exercise training has been introduced as the only practical method of providing cardioprotection against IR injury. If vibration-induced protection is nearly as effective as the exercise, it could be an alternative to exercise training, especially for those who are unable to perform traditional exercises. Delineating the mechanisms mediating vibration-induced protection against IR injury is important and could lead to the development of pharmacological or molecular approaches against cardiovascular diseases.

### Limitations of the study

One of the limitations of the present study is that it was carried out on rats. Even though the large number of animal studies have conducted and contributed much to our understanding of disease mechanisms, their findings for predicting the effectiveness of strategies in humans has remained controversial.^37^^,^^38^ Therefore, the results need to be confirmed by clinical trials in the future.

The frequency, amplitude, and the time of exposure of the subjects to vibration are important variations in clinical and experimental trials. However, due to lack of knowledge regarding optimum training protocols, the method was based on the methodology available in our laboratory. The proposed method has shown that is effective in improving health status by influencing cardiovascular disease (CVD) risk factors.^18^^,^^39^ We recommend evaluating the various vibration regimes on the IR injury in future studies.

## Conclusions

The present experimental data provide new evidence that vibration training can enhance cardiac tolerance to IR injury in an *in vivo* rat infarct model. It reduces infarct size and ischemia-induced arrhythmias and improves arrhythmia-related mortality by reducing fatal VF episodes and by facilitating spontaneous defibrillation. The finding that vibration training increases myocardial resistance to VF in this model offers experimental support for the epidemiological data associating exercise training with decreased sudden cardiac death. However, more evidence is needed in this regard.
